# Time series causal relationships discovery through feature importance and ensemble models

**DOI:** 10.1038/s41598-023-37929-w

**Published:** 2023-07-14

**Authors:** Manuel Castro, Pedro Ribeiro Mendes Júnior, Aurea Soriano-Vargas, Rafael de Oliveira Werneck, Maiara Moreira Gonçalves, Leopoldo Lusquino Filho, Renato Moura, Marcelo Zampieri, Oscar Linares, Vitor Ferreira, Alexandre Ferreira, Alessandra Davólio, Denis Schiozer, Anderson Rocha

**Affiliations:** 1 Artificial Intelligence Lab., Recod.ai, Institute of Computing, University of Campinas (Unicamp), 13083-852 Campinas, SP Brazil; 2 Center for Petroleum Engineering (CEPETRO), University of Campinas (Unicamp), 13083-970 Campinas, SP Brazil; 3 School of Mechanical Engineering (FEM), University of Campinas (Unicamp), 13083-970 Campinas, SP Brazil; 4 Group of Automation and Integrated Systems, São Paulo State University (Unesp), 18087-180 Sorocaba, SP Brazil

**Keywords:** Fossil fuels, Computational science, Computer science

## Abstract

Inferring causal relationships from observational data is a key challenge in understanding the interpretability of Machine Learning models. Given the ever-increasing amount of observational data available in many areas, Machine Learning algorithms used for forecasting have become more complex, leading to a less understandable path of how a decision is made by the model. To address this issue, we propose leveraging ensemble models, e.g., Random Forest, to assess which input features the trained model prioritizes when making a forecast and, in this way, establish causal relationships between the variables. The advantage of these algorithms lies in their ability to provide *feature importance*, which allows us to build the causal network. We present our methodology to estimate causality in time series from oil field production. As it is difficult to extract causal relations from a real field, we also included a synthetic oil production dataset and a weather dataset, which is also synthetic, to provide the ground truth. We aim to perform *causal discovery*, i.e., establish the existing connections between the variables in each dataset. Through an iterative process of improving the forecasting of a target’s value, we evaluate whether the forecasting improves by adding information from a new potential driver; if so, we state that the driver causally affects the target. On the oil field-related datasets, our causal analysis results agree with the interwell connections already confirmed by tracer information; whenever the tracer data are available, we used it as our ground truth. This consistency between both estimated and confirmed connections provides us the confidence about the effectiveness of our proposed methodology. To our knowledge, this is the first time causal analysis using solely production data is employed to discover interwell connections in an oil field dataset.

## Introduction

Understanding how a set of variables are related is becoming an important aspect in different fields of science, e.g., social and behavioral sciences, neuroscience, and econometrics. For instance, understanding a drug’s efficacy in a population^1^, if a public policy has the expected effects on society^2^, how major climate effects such as El Niño Southern Oscillation (ENSO) influence remote regions^3^, through which pathways different regions of the brain interact^4^, or the interwell connectivity for effective oil field management^5^, help comprehend the dynamics behind complex systems. To answer those questions, the standard procedure is to manipulate the value of a variable by performing a Randomized Controlled Trial (RCT). However, in many cases, it is not possible to perform such experiments because it is unethical, practically impossible, or expensive, such as performing controlled experiments to establish the causal relationship between smoking and lung cancer (see Pearl and Mackenzie^6^, Chap. 5).

The advent of Machine Learning (ML) algorithms, the increasing amount of collected data, and the increased hardware processing power now available, allow processing those data and applying complex algorithms to find causal relationships among variables (i.e., establish causality). The classical definition of causality is that $\textbf{X}$ $\rightarrow$ $\textbf{Y}$ ($\rightarrow$ meaning a causal link) if and only if an intervention or manipulation in $\textbf{X}$ affects $\textbf{Y}$^7^, where $\textbf{X}$ is known as the driver and $\textbf{Y}$ as the target. The first approach to measure the degree of associations among variables is to compute the correlation. However, the correlation is symmetrical and does not take into account the directionality of the relationship. On the other hand, causation is asymmetrical and provides the directionality of the relation among the variables^8^.

Why do we need to establish causality and not only work on establishing statistical associations? One major reason is that causality enables us to predict the underlying dynamics of the system, pinpointing which variables can be intervened to achieve the desired output^9^ through a causal model. The ladder of causation (see Pearl and Mackenzie^6^, Chap. 1) is defined by three steps that goes from simple data association to hypothetical imaging scenarios (“what if I had done”). This paper focuses on the first step, establishing the associations between variables in a dataset; current ML algorithms are also on this rung since they only rely on data and are thus unable to operate in unseen scenarios. In our main application, we have the challenging problem of production in Pre-Salt fields. In this problem, identifying the causal links between the injector and producer wells is critical, as it influences operational decisions and helps to optimize production. Once the associations are discovered, we can go to the second rung called *interventions*: Given a relationship between $\textbf{X}$ and $\textbf{Y}$, this level answers questions such as “what would be $\textbf{Y}$ if I intervene $\textbf{X}$, does $\textbf{Y}$ happen?”. Finally, the last rung is called *counterfactuals* and answers questions such as “Was it $\textbf{X}$ that caused $\textbf{Y}$?” or “What if $\textbf{X}$ had not occurred?”^10^.

Most efforts to infer causal structures from observations are carried out through the following approach: one assumes a multivariate distribution and then uses observational data to acquire knowledge of the parametric model or estimate the statistics. Then, the model’s suitability to the observed data and adherence to the imposed constraints is evaluated, to determine whether the assumed causal framework accurately describes the observed phenomena.

Inspired by the Ladder of Causation, Peyrard and West^11^ propose metrics based on the observational, interventional, and counterfactual distances of two different causal models, establishing a way of comparing the models based on the causal distribution induced by them, instead of their graphical structures, as usually found in the literature.

Aiming to distinguish between accidental associations and robust causal associations that support counterfactual reasoning, Barbero and Sandu^12^ propose an approach called causal team, which combines team semantics and structural equations models. This framework consists of a set of assignments over a given collection of variables expanded with a collection of structural equations associated with some of its variables, being able to account for associations and evidential, observational reasoning and to account for interventions and support counterfactual claims.

To help ML-based agents to climb the Ladder of Causation and achieve counterfactual reasoning, Moruzzi^13^ points out that such agents must incorporate compositionality and sparsity, to guarantee that the agent can explain new data observed in previously unknown scenarios, identifying connections between different variables when determining which variables are responsible for changes in the scenario. The author points out that promising strategies for this include the representation of variables in sparse factor graphs, the meta-learning approach called Recurrent Independent Mechanisms, and the use of neuro-symbolic models.

Why is causality hard to establish? When attempting to assess causality, we must ensure that every possible variable that might be involved in the system’s dynamic must be considered to avoid spurious correlations. In a real-world scenario, this condition is rarely accomplished: There might be unobserved *confounders* (when investigating a potential causal-and-effect relationship, a *confounding variable* is a third variable that influences both the supposed cause and the supposed effect), the data are scarce, and few variables may change simultaneously and influence the outcome we observe, either directly or indirectly. This lack of control over the system’s dynamics makes it even harder to claim that a causal relationship exists, which leads researchers to depend highly on the opinion of experts in the area to have more confidence in the findings.

One of the pitfalls of causal analysis is the so-called Yule–Simpson paradox ^14,15^ which can occur when a confounder is omitted, causing a measure of association between two variables to change from positive to negative. Kuang et al.^16^ propose that to avoid the Yule–Simpson paradox in purely observational approaches it is necessary to validate the assumptions needed for causal inference using domain knowledge, and to additionally conduct a sensitivity analysis to identify potential violations of these assumptions.

In this paper, we focus on time series (ordered sequences of values), in which causal discovery aims to establish the causal links, including the time lags (time-lagged causal discovery). Lags tell us the time delay between a cause and the occurrence of its effect. Related to time series, ML algorithms have been used for classification^17^, clustering^18^, and forecasting^19,20^, but little work has been done to employ ML to infer causal relationships in time series (see Moraffah et al.^21^ for a full review in the subject). Firstly, we tested our method on a weather dataset that is publicly available on CauseMe.net. Additionally, we focus on applying our algorithm to the challenging context of the Pre-Salt field, encompassing both public synthetic and private real fields. Given the unique and demanding nature of the Pre-Salt field, our method has been specifically designed to address its robustness requirements, considering the field’s renowned for its complex geology. This application highlights the relevance and effectiveness of our method in a real-world scenario, contributing to the optimization of production, reservoir management, and overall performance of the Pre-Salt field.

We organize the paper into the following sections: “Time series causality assessment” section details causality assessment; “Proposed methodology” section describes our proposed methodology to assess causality in time series; “Applications” section presents applications of causal discovery applied in two different domains—oil field production data and climate; finally, “Conclusions and future work” section outlines final remarks discussing our main findings and future work.

## Time series causality assessment

This section formally sets out the problem of causality inference in time series, and its main challenges, and presents the most explored methods in the literature for its solution.

### The causal relationships

**Figure 1:**
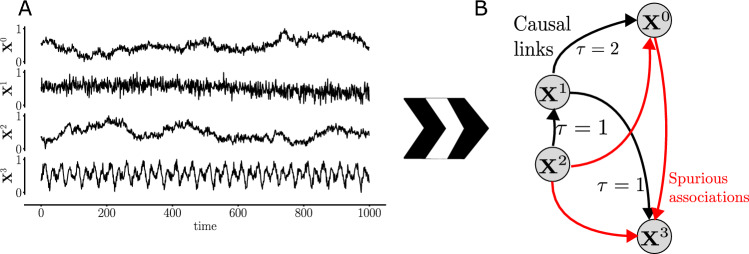
Given a set of multivariate time series (**A**), causal discovery aims to estimate the underlying causal dependencies, including the time lags $\tau$ (labels on the edges) (**B**). Spurious associations can appear due to either common drivers, e.g., $\textbf{X}^{0}\leftarrow \textbf{X}^{1}\rightarrow \textbf{X}^{3}$ (correlation between $\textbf{X}^{0}$ and $\textbf{X}^{3}$) or indirect paths, e.g., $\textbf{X}^{2}\rightarrow \textbf{X}^{1}\rightarrow \textbf{X}^{0}$ (correlation between $\textbf{X}^{2}$ and $\textbf{X}^{0}$) or $\textbf{X}^{2}\rightarrow \textbf{X}^{1}\rightarrow \textbf{X}^{3}$ (correlation between $\textbf{X}^{2}$ and $\textbf{X}^{3}$). Adapted from^4^.

Given a dataset $\mathscr {X}$ with *N* time series of the same length *T*, i.e., $\mathscr {X}=\{\textbf{X}^{1}, \textbf{X}^{2}, \dots , \textbf{X}^{N}\} \in \mathbb {R}^{N\times \,T}$, we want to discover the causal relationships between all *N* time series in $\mathscr {X}$ and the time lag between a cause and the occurrence of its effect^8^. Figure 1 shows a sketch of how the process of causal discovery in time series works: From a set $\mathscr {X}$ of variables, possible causal links are tested from variable $\textbf{X}^{l}$ to $\textbf{X}^{k}$, $k,l=1, 2, \dots , N$ (including links from the past of $\textbf{X}^{k}$ itself, i.e., $l=k$). This allows us to reconstruct the underlying causal dependencies (solid black arrows), discard spurious associations (red arrows; when the correlation is significant, but there is no causal dependence), and the time dependence in the system because of the lags $\tau$. The main challenges faced by any method that seeks causal discovery are:

1. Distinguish direct from indirect causes. In Fig. 1, variable $\textbf{X}^{1}$ directly affects both $\textbf{X}^{0}$ and $\textbf{X}^{3}$, but $\textbf{X}^{2}$ has an indirect effect on $\textbf{X}^{0}$ through $\textbf{X}^{1}$ ($\textbf{X}^{1}$ acts as a mediator).
2. Distinguish instantaneous causal effects, i.e., $\tau =0$.
3. Deal with *confounders*: In Fig. 1, $\textbf{X}^{1}$ is a common cause for both $\textbf{X}^{0}$ and $\textbf{X}^{3}$, which explains the spurious correlation between $\textbf{X}^{0}$ and $\textbf{X}^{3}$.

### Related work

Because of the vast amount of time series data in different areas, several efforts have been made to tackle causality in this type of data. Over the last decades, different methods have been proposed to assess this challenge. Those methods can be categorized as follows^8,21^:

#### Methods based on Granger causality

One of the first approaches to tackle causality in time series was suggested by Granger et al.^22^. Given two time series, $\textbf{X}$ (possible driver) and $\textbf{Y}$ (target), it says $\textbf{X}$ Granger-cause $\textbf{Y}$, if and only if considering the past of $\textbf{X}$, improves the forecasting of $\textbf{Y}$ at time *t*. It means that $\textbf{X}$ contains unique information about $\textbf{Y}$, not contained in $\textbf{Y}$’s past. Mathematically, this can be expressed as a Vector Autoregressive Models (VAR):

1 $$\begin{aligned} \textbf{Y}_{t} = \sum _{\tau =1}^{\tau _{\textrm{max}}}a_{\tau }\textbf{Y}_{t-\tau } + \sum _{\tau =1}^{\tau _{\textrm{max}}}b_{\tau }\textbf{X}_{t-\tau } + \eta _{t}, \end{aligned}$$

in which $\tau _{\textrm{max}}$ is the maximum lag into the past that we want to consider.

If $\textbf{X}$ Granger-cause $\textbf{Y}$, it means some values $b_{\tau }$ are not zero. The main drawback of this approach is that it assumes a linear relationship among the variables, and it might not be suitable for every case in which we want to establish causal relationships. Some extensions of Granger causality applied to multivariate time series can be found in Haufe et al.^23^ and Siggiridou and Kugiumtzis^24^.

#### Conditional independence-based methods

Those methods test conditional independence relations between variables and their past, i.e., it checks for conditional independence between $\textbf{X}_{t-\tau }^k$ and $\textbf{X}_{t}^{l}$ given the past of those variables (time series). Some assumptions are made^4,7^:

*Time-order*: Cause precedes effects.
*Causal sufficiency*: All direct common drivers are observed.
*Causal Markov Condition*: If two variables are not connected in the causal graph conditioned on their parents (see Spirtes et al.^25^, Chap. 3), they are conditionally independent.
*Faithfulness*: If two variables are independent given a subset of variables, then they are not connected by a causal link in the graph.

Applications of this class of methods can be found in Chu & Glymour^26^ and Runge^27^.

#### Structural equation-based models

Structural Equation Models (SEM) is a collection of statistical techniques to examine relationships between one or more independent variables and dependent variables^28^. Mathematically, it can be expressed as $\textbf{X}:=f(\textbf{V}, \varepsilon _{\textbf{X}})$, i.e., where each substantive variable $\textbf{X}$ is a function of other variables $\textbf{V}$, and a unique error term $\varepsilon _{\textbf{X}}$^29^. The usage of the assignment operator ($:=$), rather than an equality operator, is because the equations must be interpreted causally: Manipulating a variable *V* can lead to a change in *X*. The substantive variables are the ones of interest but are not necessarily observed in their totality. An application using SEM applied to time series causal discovery, called Time Series Models with Independent Noise (TiMINo), can be found in Peters et al.^30^.

#### Deep learning-based models

Traditional time series causal discovery methods assume some linearity in the time series, like Granger causality. However, real-world cases can be nonlinear, so those methods are unsuitable for such scenarios. Transfer Entropy is an alternative suitable for linear and nonlinear scenarios, but it is difficult to implement and sensitive to parameter selection^31^. To overcome those issues and take into account nonlinearity, some approaches have been proposed to model time series using neural network architectures like Multi Layer Perceptron (MLP), Recurrent Neural Network (RNN) ^32^ and Convolutional Neural Networks (CNNs) ^8^. Usually, the inputs to those Deep Learning (DL)-based frameworks are the past lags of every time series (drivers), and the outputs are the future values of a single time series (target). CNNs use an attention mechanism to provide interpretability in the model. The attention mechanism coefficients can be interpreted as the model feature importance^33^.

A summary of common causal discovery methods applied to time series is shown in Nauta et al.^8^ Each method includes a description on whether it deals with aspects like *confounders*, *hidden confounders* and the necessity of stationary data, among other aspects. The main drawbacks of those methods relate to the existence of *hidden confounders* in the data as well as the need for stationary time series, since the methods could not work as expected when fed with this kind of data.

## Proposed methodology

The general idea to assess causality is to be able to pinpoint which predictors (variables and their lags) the algorithm considers the most when predicting an output, for instance, through the coefficients per lag in the Granger causality approach (see “Methods based on Granger causality” section) or the usage of attention mechanisms in DL-related algorithms (see “Deep learning-based models” section). This section presents our proposed methodology, which we call Aleph, in honor of Latin American writer Jorge Luis Borges, who saw in the causal flow the foundation of both the world and the narrative activity, and who architected many of the short stories of his masterpiece, “The Aleph”, from this perspective^34^.

In our proposed methodology, we first define which variables (possible drivers) causally affect a target. Once a set of $\mathscr {D}$ drivers known to causally affect the target is discovered (“Time series causal discovery” section), we proceed to assess which are the most significant features (lags) based on its feature importance (“Assessing feature significance” section), i.e., the discovery of the lags of influence from every driver to the target. For the first step, any regression method could be employed but, for the second one, we need ML algorithms that quantify the importance of each feature when predicting the output. Ensemble algorithms like Random Forest (RF) or CatBoost provide this information on feature importance. Therefore, we employ them to identify which predictors the model pays attention to the most when generating the forecasting and, from that information, we reconstruct the causal path (see Fig. 1).

**Figure 2:**
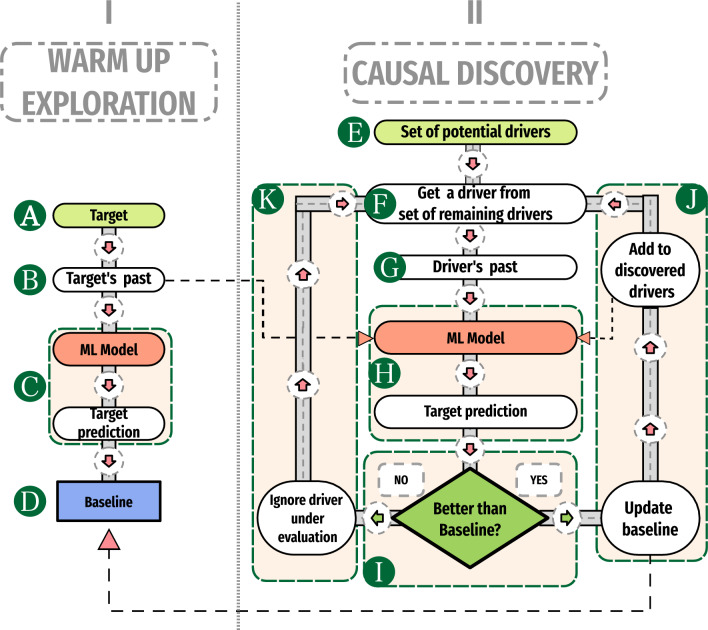
Pipeline used to perform causal discovery. Firstly, a model is built considering only past-lagged values of the target itself, which defines the forecasting baseline (I). The second step is to build a model considering information from both the target and a potential driver that may causally affect the target (II). If there is an improvement in the forecasting, the driver is kept as a predictor (added to the list of discovered drivers, and the baseline is updated). When a new potential driver is tested, information from the already tested drivers that causally affect the target (discovered drivers) is also included.

The general pipeline we propose to perform causal discovery is shown in Fig. 2. The pipeline comprises two major steps:

- Establish a baseline by considering only the target’s past values (block I) to predict the target’s current value; and
- Using information from potential drivers in an attempt to improve the forecasting (block II).

**Figure 3:**
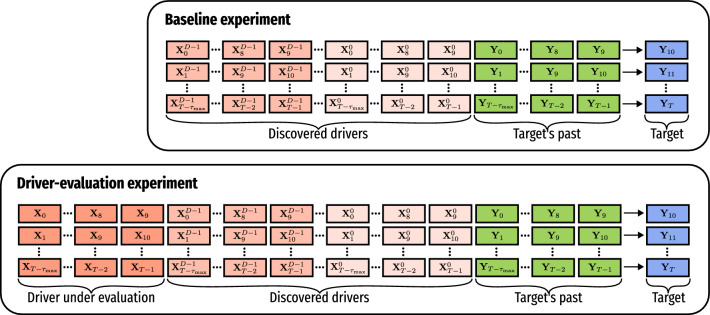
Representation of how feature vectors are defined through a sliding window to assess a causal link from a driver. The first set of features is defined using $\tau _{\textrm{max}}{=}10$ past-lagged values—at first from the target $\textbf{Y}$ only, then also with all the *D* already discovered drivers $\textbf{X}^{0}, \dots , \textbf{X}^{D-1}$—to predict the current target’s value $\textbf{Y}_{t}$ (Baseline experiment). The second set of features is defined considering $\tau _{\textrm{max}}$ past-lagged values from the target variable $\textbf{Y}$ as well as from discovered drivers $\textbf{X}^{0}, \dots , \textbf{X}^{D-1}$ along with values from driver $\textbf{X}$ under evaluation to predict the current target’s value $\textbf{Y}_{t}$ (Driver-evaluation experiment). When the forecasting of the current target’s value $\textbf{Y}_{t}$ significantly improves over the baseline by considering data from a new driver $\textbf{X}$, we consider that driver causally affects the target.

The pipeline works as follows (and following the blocks identified by capital letters in Fig. 2):

A: Given a set of variables on which we want to perform causal discovery, we select one to be considered as a potential target, i.e., the one to which causal relationships will be assessed.
B: Using past data from the potential target chosen in step  (A), we define feature vectors with past-lagged values from the potential target (see Fig. 3).
C: An ensemble ML model is trained based on the features defined in step (B) and used to predict the target’s current value (more details in “Time series causal discovery” section; see Fig. 3).
D: An initial *baseline* is established as the evaluation of a metric on the forecasting of step (C) in comparison with the ground truth.
E: A *set of potential drivers* that might causally affect the potential target is defined.
F: If the *set of potential drivers* defined in step (E) is not empty, a driver is selected for consideration and removed from that set. Otherwise, the result of the pipeline is the *set of discovered drivers* incremented through step (J)
G: Using past data from the potential driver chosen in step (F), we define feature vectors with past-lagged values from the potential driver.
H: Feature vectors from the potential target of step (B), from already *discovered drivers* through step (J), and from the potential driver under consideration chosen in step (F), are used to predict the target’s current value (see Fig. 3).
I: The metric is evaluated based on the forecasting of the target’s current value by the model in step (H) and compared against the *baseline*. If the forecasting of the model of step (H) is better than the *baseline*, we go through step (J); otherwise we go through step (K). In either case, we return to step (F).
J: We state the driver selected in step (F) causally affects the target under evaluation, then the *baseline* is updated with the metric evaluated in step (I) and the driver under consideration is added to the *set of discovered drivers*.
K: We consider that the driver under consideration does not causally affect the target, so we ignore it.

The main advantages of our approach to assessing causality are that:

(i) It can look at causal relationships in nonlinear data.
(ii) Handle multivariate and continuous time series.
(iii) Take care of *confounders* (see “Step-by-step toy example” section).
(iv) Leverage the *feature importance* given by the ensemble models to pinpoint the delay (lag) between cause and effect.

We applied our method in datasets (both simulated and real) from different areas to test the applicability of our approach.

In-depth details are shown in “Time series causal discovery” , "Assessing driver significance" and “Assessing feature significance” sections. A toy example was devised and each step taken to perform causal discovery (following the pipeline in Fig. 2) is shown in “Step-by-step toy example” section.

### Time series causal discovery

Numerous ML techniques have been employed to determine causality in time series^8,35,36^. The idea behind those approaches is similar to the one of Granger causality, to test whether adding information from the past of possible drivers improves the forecasting of future values of the target. The advantage of ML models is that they are not restricted to variables with linear relationships (as some cases of Granger causality).

We employed two models for regression based on ensembles: RF^37^ and CatBoost^38^. Both models are employed here as regressors for predicting continuous values. We first used them to evaluate if a variable significantly aids the regression in terms of performance, therefore considering that variable as the target variable’s driver. Given the variables $\textbf{X}$ and $\textbf{Y}$, to determine if $\textbf{X}$ drives (causally affects) $\textbf{Y}$, we define the past values of both variables as features $\textbf{X}_{t-\tau _{\textrm{max}}}, \dots , \textbf{X}_{t-1}, \textbf{Y}_{t-\tau _{\textrm{max}}}, \dots , \textbf{Y}_{t-1}$, having $\textbf{Y}_{t}$ as target, as depicted in Driver-evaluation experiment of Fig. 3 section. Similarly, we proceed to build features from $\textbf{Y}$ as well with feature vectors $\textbf{Y}_{t-\tau _{\textrm{max}}}, \dots , \textbf{Y}_{t-1}$ also with target $\textbf{Y}_{t}$, as depicted in Baseline experiment of Fig. 3. In any case, the goal is to predict $\textbf{Y}$’s current value $\textbf{Y}_{t}$ by using that past information. If information from $\textbf{X}$ (lagged values) improves the forecasting of $\textbf{Y}_{t}$ compared to the baseline, and that improvement is *significant* according to the method described in “Assessing driver significance” section, we consider that $\textbf{X}$ causally affects $\textbf{Y}$. Therefore, $\textbf{X}$ is included in the list of *discovered drivers* before the evaluation of a new driver.

One might wonder if the order of consideration of drivers from the set of possible ones matters. As we will see, for the oil field production data, we considered the order of the drivers based on the geographical distance from the target (the closer, the first to be considered). For the climate dataset, we considered an arbitrary order based on the order of specification of the variables in the dataset. Although we did not evaluate the influence of the order in those datasets, we experimented with the synthetic case of “Step-by-step toy example” section. In that case, there are eight possible drivers which lead to a permutation of 40320 possible orders of consideration. From those, we randomly tested 100 cases and observed that our method obtains the same ground truth set of drivers in most of them, indicating that the order does not seem to matter much.

### Assessing driver significance

As we previously mentioned, it is not enough that a variable $\textbf{X}$ improves the forecasting of $\textbf{Y}_{t}$ to have it considered as a driver. The key point in our approach to assessing causality is if there is a *significant improvement* in predicting the target’s value by adding information from potential drivers. To quantify the quality of the forecasting, we can use any metric commonly used for regression. To compute the significance, as we evaluate a new driver, we perform a random shuffling of the features coming from that driver. We selected the shuffling approach over other surrogate techniques due to its simplicity and easiness of application to decompose the temporal relationships between driver and the target in an unsupervised way. Moreover, this formulation is less computationally costly. We shuffle the features, train a model, make a forecasting on the testing set, and retrieve the quality of the forecasting (metric), repeating the process *n* times. We end up having a set $\mathscr {M}=\{m_{1}, m_{2}, \dots , m_{n}\}$ of values for the metric due to the shuffling process and, additionally, we have the metric value $m_{\textrm{real}}$ we obtain when training and testing the model without shuffling the data. The goal is to assess the statistical significance of $m_{\textrm{real}}$ against the distribution given by $\mathscr {M}$ at a significance level $\alpha$. We fit the data in $\mathscr {M}$ with a statistical distribution and compute the probability of $m_{\textrm{real}}$ being obtained by chance. If this probability is less than $\alpha$, we consider the driver causally affecting the target.

### Assessing feature significance

Similarly, we proceed to assess the significance of each feature. Once we establish the set of drivers $\mathscr {D}=\{\textbf{X}^{1}, \textbf{X}^{2}, \dots , \textbf{X}^{D}\}$ which causally affect the target, we shuffle each feature from the drivers in $\mathscr {D}$ individually, keeping the other features unchanged. We train the model and retrieve the feature importance provided by the ensemble model, repeating the process *n* times and building the set $\mathscr {F}_{\textbf{X}_{t-\tau }^{d}}=\{m_{1}, m_{2},\dots , m_{n}\}$ per driver *d*, per feature/lag $\tau$. Similarly, as for assessing driver significance, we fit the data in $\mathscr {F}_{\textbf{X}_{t-\tau }^{d}}$ with a statistical distribution and compute the probability of $m_{\textrm{real}}$ of the feature without shuffling being obtained by chance, considering a significance level $\alpha$.

### Combining time series

Depending on the research area we seek to establish causality, time series could be very similar among them. One of the reasons is that the system is constantly controlled (like the case of the oil industry that, for technical reasons, values of certain variables must be kept within a given range). Let us define as an *entity* a driver or target for which we have more than one kind of measurement (information from more than one variable from the same entity). It may occur that time series for the same variable and from different entities are similar, for instance, in terms of Pearson’s correlation coefficient. Since we aim to obtain a unique time series representation per entity, we propose combining the time series from the same entity into one in order to circumvent this drawback.

We propose using Uniform Manifold Approximation and Projection (UMAP) ^39^ to obtain time series representation per entity. In this approach, we perform a random grid-search experimenting with different combinations of the UMAP’s hyperparameters *number of neighbors*, *minimum distance* and *spread*. The same setup is applied to every available time series per entity. To determine which UMAP’s hyperparameters combination leads to the best results in terms of establishing causal relationships, we used the output of the combination as input in the pipeline defined in Fig. 2, find the causal drivers and compare them against a ground truth (if available).

**Figure 4:**
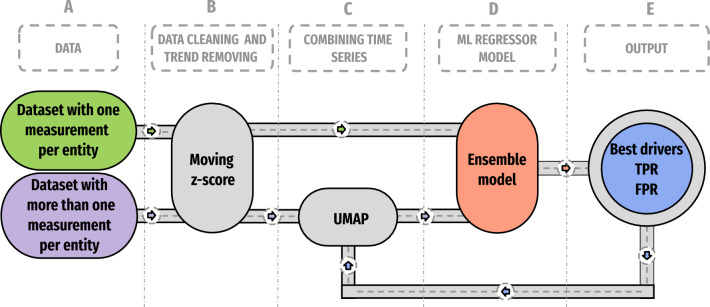
Pipeline for processing and combining (if necessary) time series prior to using them as drivers or targets. (**A**) Data selection is performed considering the best time windows. (**B**) Data cleaning and trend removal are applied to the time series on the selected time windows only to keep variability. (**C**) For the specific case of more than one measurement per entity, time series from the same entity are combined into one using UMAP. (**D**) Time series from target and drivers are used to assess causality; the pipeline given in Fig. 2 fits in this block. (**E**) The outcome of the ensemble model provides the answer if the driver causally affects the target. If so, the causal driver is compared against the ground truth (if available) to create the Positive Rate (TPR) and False Positive Rate (FPR) metrics.

In Fig. 4, we show the pipeline followed to combine time series (if needed). Given a dataset with more than one measurement per entity and, if we want to obtain a unique time series representation, we proceed as shown in the lower block. Time series are filtered and those from the same entity are combined into one. The output is then used as a driver or target in Fig. 2. In case the dataset has only one measurement per entity, filtering is applied to the time series and is ready to be used as a target/driver.

**Figure 5:**
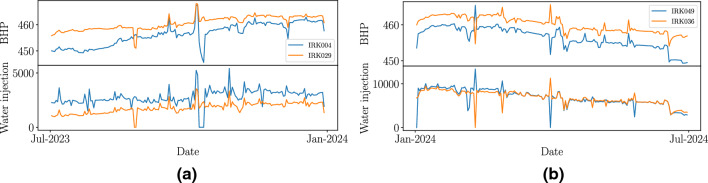
Example of time series similarity. Panels (**a**) and (**b**) show examples of both BHP and water injection rate, respectively, for two different pairs (one pair per panel) of injector wells in a simulated dataset. In those plots, we can see how similar the water injection time series are for each pair of compared wells. A similar degree of similarity can be seen in the BHP’s time series and this similarity is quantified through Pearson’s correlation coefficient. Similar behavior is found in time series from a real oil field related dataset.

In this paper, we used this approach to combine time series for oil field related datasets: Time series among various injector wells are correlated to each other because the system is human-controlled. Fig. 5 shows two examples of similarity among pairs of injectors’ time series, in which similarity is quantified by calculating Pearson’s correlation coefficient. The correlation coefficients are high: $\sim 0.8$ for the Bottom Hole Pressure (BHP)’s time series and $\sim$ $0.6$ for water injection. In Fig. 5a is shown both for the time series of BHP and water injection from injectors IRK004 and IRK029; similarly applies to injectors IRK049 and IRK036 shown in Fig. 5b. This high similarity prevents establishing any causal connection between injectors and producers because it is hard to differentiate the injectors’ time series from each other.

## Step-by-step toy example

To test our algorithms preliminarily (see “Proposed methodology” section) and, as a proof of concept, we devised a synthetic example to perform time series causal discovery.

### Example of establishing causal relationships

In Eq. (2), we show the mathematical definition—following a VAR model—of each time series and which ones are causally affected (namely $\textbf{X}^{1}$, $\textbf{X}^{3}$ and $\textbf{X}^{7}$) by the others. For this example, we consider as targets only $\textbf{X}^{1}$ and $\textbf{X}^{7}$. The reason for this mathematical construction is to test if we are able to distinguish direct and indirect effects from the drivers: $\textbf{X}^{3}$ is affected by $\textbf{X}^{2}$, meaning $\textbf{X}^{2}$ has an indirect effect (through $\textbf{X}^{3}$) on $\textbf{X}^{1}$, we want to test if our method can pinpoint $\textbf{X}^{3}$ as a causal driver, but not $\textbf{X}^{2}$ on $\textbf{X}^{1}$. For each time series $\textbf{X}$, the subindex $t-\tau$ ($\tau$ being an integer) means the lag dependence (past values) of the time series’ current value $\textbf{X}_{t}$. As shown in Eq. (2), most of the time series depend on themselves, but for those time series being causally affected by the others, the dependence is with both itself and the other time series.

2a $$\begin{aligned} \textbf{X}_{t}^{0} = 0.3\textbf{X}_{t-4}^{0} + 0.4\textbf{X}_{t-2}^{0} +\eta ^{0}, \end{aligned}$$

2b $$\begin{aligned} \varvec{X}_{t}^{1} & = 0.4\textbf{X}_{t-4}^{1} + 0.5\textbf{X}_{t-4}^{3} + 0.3(\textbf{X}_{t-2}^{3})^{2} + 0.4\textbf{X}_{t-5}^{0} + 0.3(\textbf{X}_{t-9}^{0})^{2} + 0.3(\textbf{X}_{t-1}^{4})^{2} + 0.4\textbf{X}_{t-7}^{4}\nonumber \\ & \quad + 0.4\textbf{X}_{t-3}^{6} + 0.6\textbf{X}_{t-8}^{6} + \eta ^{1}, \end{aligned}$$

2c $$\begin{aligned} \textbf{X}_{t}^{2}= 0.4\textbf{X}_{t-1}^{4} + 0.2\textbf{X}_{t-2}^{2} + \eta ^{2}, \end{aligned}$$

2d $$\begin{aligned} \textbf{X}_{t}^{3}= 0.1\textbf{X}_{t-3}^{3} + 0.3\textbf{X}_{t-2}^{3} + 0.4\textbf{X}_{t-6}^{2} + \eta ^{3}, \end{aligned}$$

2e $$\begin{aligned} \textbf{X}_{t}^{4}= 0.3\textbf{X}_{t-8}^{4} + 0.3\textbf{X}_{t-1}^{4} + \eta ^{4}, \end{aligned}$$

2f $$\begin{aligned} \textbf{X}_{t}^{5}= 0.5\textbf{X}_{t-5}^{5} + 0.2\textbf{X}_{t-9}^{5} + \eta ^{5}, \end{aligned}$$

2g $$\begin{aligned} \textbf{X}_{t}^{6}= 0.4\textbf{X}_{t-8}^{6} + 0.4\textbf{X}_{t-9}^{6} + \eta ^{6}, \end{aligned}$$

2h $$\begin{aligned} \textbf{X}_{t}^{7}= 0.5\textbf{X}_{t-9}^{7} + 0.8\textbf{X}_{t-1}^{4} + 0.7\textbf{X}_{t-6}^{4} + 0.4\textbf{X}_{t-7}^{5} + 0.6\textbf{X}_{t-8}^{5} + 0.9\textbf{X}_{t-1}^{8} + 0.7(\textbf{X}_{t-2}^{8})^{2} + 0.7\textbf{X}_{t-3}^{9} + 0.8\textbf{X}_{t-4}^{9} + \eta ^{7}, \end{aligned}$$

2i $$\begin{aligned} \textbf{X}_{t}^{8}= 0.1\textbf{X}_{t-1}^{8} + 0.1\textbf{X}_{t-2}^{8} + \eta ^{8}, \end{aligned}$$

2j $$\begin{aligned} \textbf{X}_{t}^{9}= 0.3\textbf{X}_{t-7}^{9} + 0.5\textbf{X}_{t-8}^{9} + \eta ^{9}. \end{aligned}$$

**Figure 6:**
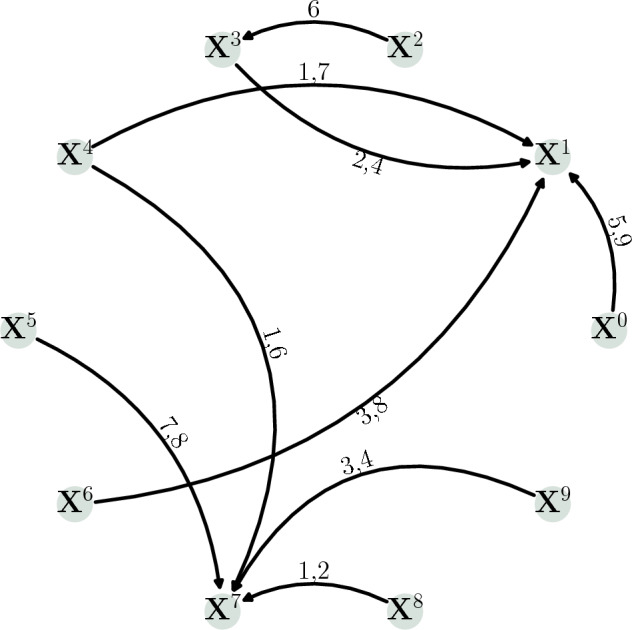
Graph of connections for the synthetic example given by Eq. (2). The label on the edge between every two nodes means the lag dependence of the target related to the driver.

**Figure 7:**
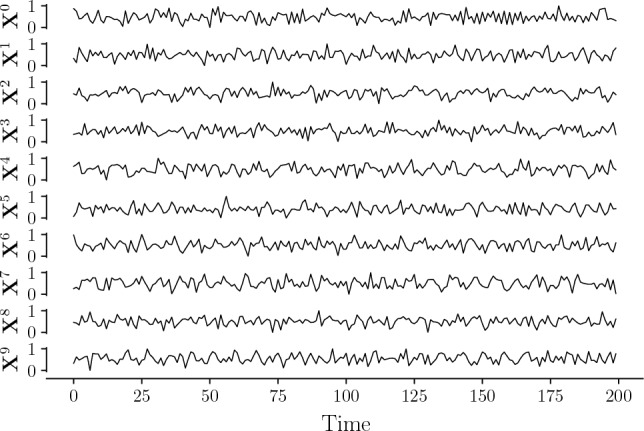
Representation of time series given by Eq. (2). Each time series was generated considering a VAR model of a different order.

**Figure 8:**
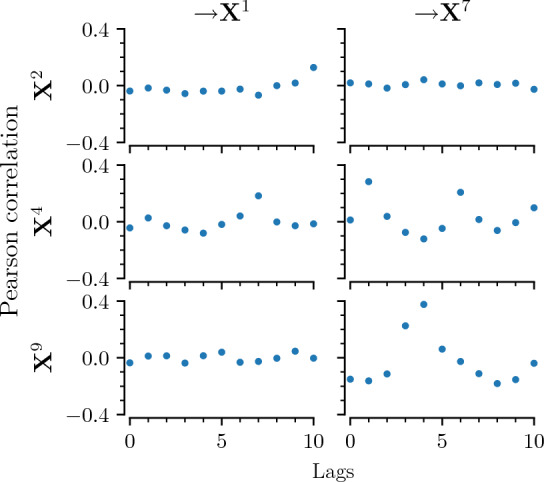
Pearson correlation at different lags for a few of the time series given by Eq. (2). There is no strong correlation (close to 1) of either $\textbf{X}^{1}$ or $\textbf{X}^{7}$ with some of their drivers (rows), even though we know by definition in Eq. (2) there is an influence from the drivers. This shows that, even with a low correlation between drivers and the target, this does not mean the absence of causal relationships.

Figure 6 shows the graph with the ground truth of the causal links between the time series, where the label on each edge shows the dependence lags between the target and the driver. Figure 7 presents the time series given by Eq. (2); for visualization, a short number of data points were generated. This figure does not show seasonality or trend in the time series. Figure 8 shows a few cases of comparison between time series by computing Pearson correlation. Each panel shows the Pearson’s cross-correlation coefficient between the time series $\textbf{X}^{1}$ or $\textbf{X}^{7}$ (columns) with itself or potential drivers (rows) at different lags: There is no strong evidence (looking only at correlations) of causal relationships among the variables, even if we know they exist by definition.

**Figure 9:**
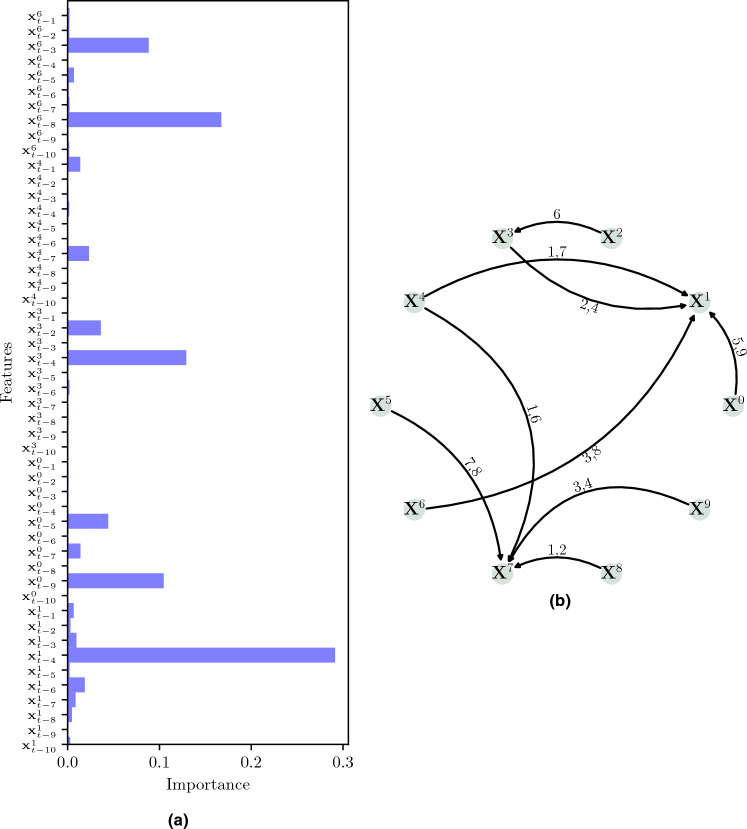
Panel **(a)** shows the feature importance for synthetic example with $\textbf{X}^{1}$ as target. The features (lags) with higher importance correspond to the drivers’ lags influencing the target. For instance, the feature $\textbf{X}_{t-9}^{0}$ means the variable $\textbf{X}^{0}$ causally affects the variable $\textbf{X}^{1}$ nine timestamps later. To ease comparison, we added panel **(b)** that shows the ground truth for the toy example.

Our algorithm applied to this synthetic example works as described below (mapping the steps in Fig. 2):

A: Given the set of variables $\{\textbf{X}^{0}, \textbf{X}^{1}, \dots , \textbf{X}^{9}\}$, we select $\textbf{X}^{1}$ as potential target (the same procedure applies for $\textbf{X}^{7}$ when it is considered as target).
B: Using past data from $\textbf{X}^{1}$, we define feature vectors with past-lagged values $\textbf{X}_{t-\tau }^{1}$, $0<\tau \le \tau _{\text {max}}$, in which $\tau _{\textrm{max}}{=}10$.
C: An ensemble ML model is trained based on the features $\textbf{X}_{t-\tau }^{1}$ and used to predict $\hat{\varvec{X}}_{t}^{1}$ as being the target’s current value $\varvec{X}_{t}^{1}$.
D: An initial *baseline* is established as the evaluation of a metric on the forecasting $\hat{\varvec{X}}_{t}^{1}$ in comparison with the ground-truth $\varvec{X}_{t}^{1}$.
E: A *set of potential drivers* $\mathscr {D}^p=\{\textbf{X}^{0}, \textbf{X}^{2}, \dots , \textbf{X}^{9}\}$ that might causally affect $\textbf{X}^{1}$ is defined.
F: If $\mathscr {D}^p$ is not empty, a driver $\textbf{X}^{d}$ is selected for consideration and removed from that set, otherwise the result of the pipeline is the set $\mathscr {D}$ of *discovered drivers* incremented through step (J).
G: Using past data from $\textbf{X}^{d}$, we define feature vectors with past-lagged values $\textbf{X}_{t-\tau }^{d}$, $0<\tau \le \tau _{\text {max}}$.
H: Features vectors $\textbf{X}_{t-\tau }^{1}$, features vectors from already *discovered drivers* in $\mathscr {D}$, and feature vectors $\textbf{X}_{t-\tau }^{d}$ are used to predict $\hat{\varvec{X}}_{t}^{1}$.
I: The metric is evaluated based on $\hat{\varvec{X}}_{t}^{1}$ against $\varvec{X}_{t}^{1}$ and compared with the *baseline*. If the forecasting of the model of step (H) is better than the *baseline*, we go through step (J), otherwise we go through step (K). In either case, we return to step (F).
J: We state $\textbf{X}^{d}$ causally affects $\textbf{X}^{1}$, then the *baseline* is updated with the metric evaluated in step (I) and $\textbf{X}^{d}$ is added to the set $\mathscr {D}$ of *discovered drivers*.
K: We consider that $\textbf{X}^{d}$ does not causally affect $\textbf{X}^{1}$, so we ignore it.

Once every driver in $\mathscr {D}^p$ is tested and, if $\mathscr {D}$ ends up non-empty, the features from the drivers in $\mathscr {D}$ and from the target itself are tested for significance, as is explained in “Assessing feature significance” section, to determine the most significant features (lags) as shown in Fig. 9. In that figure, we can see that the more prominent features match the lags we defined in Eq. (2) and shown in Fig. 6, resulting in a sensitivity and specificity of 1.00 for this target, and the same for all target connections in the toy example.

## Applications

We tested our algorithms in different datasets to check causal relationships between time series. In “Climate” section, we show the results of applying causality tests to weather-related time series. “Synthetic oil production field” section provides the results of applying causality in time series related to a synthetic reservoir. Finally, in “Actual oil production field” section, we present results of applying causality to a real production dataset from a Pre-Salt oil field.

Some data pre-processing is required before used it as input in the causal pipeline:

- The time series must be transformed to be stationary.
- From the toy example, we ran some tests varying the number of data points and concluded that below 500 data points, the method is not able to detect completely every link. For the oil-related dataset, in the best scenario, we have $\sim$ 360 data points, which is not a significant dataset’s size for an ML algorithm. We increased our dataset’s size through augmentation and interpolation algorithms.
- We applied interpolation to fill in missing data in the oil-related datasets. From discussion with oil-related experts, we defined a threshold of maximum 20 days of missing data in order to apply interpolation. If periods of missing data were longer, a time window over a different period was defined for applying causal analysis.

### Climate

To test our causality methods on data from different fields of science, we use the data available by Runge et al.^40^. From the platform CauseMe.net, we chose dataset TestWEATHnoise which has four sub-datasets that define the number of variables (*N*) and length (*T*, number of observations) in each file (see “Data availability” section); we employed the sub-dataset with $N{=}5$ and $T{=}2000$. The description of this dataset provided by the authors says:

These weather-type datasets feature typical weather data challenges (autocorrelation, nonlinearity, and time delays) combined with two common computational/statistical challenges, namely high dimensionality and short to large sample sizes. This data is additionally contaminated with observational noise.

To validate our algorithm in this dataset, the platform requires that the results must be submitted in an already defined format. To our knowledge, the ground truth is not available for download. Our algorithm was applied to each file in the dataset we chose and results were generated for uploading. Once we submit the results, the platform computes the Area Under the ROC Curve (AUC) as a metric to quantify the quality of our method in pinpointing real connections. We obtained an AUC of 0.62 on the dataset TestWEATHnoise_N-5_T-2000. More information on the dataset can be found in Runge et al.^41^. Methods proposed by Weichwald et al.^42^, like slarac (Subsampled Linear Auto-Regression Absolute Coefficients) and selvar (Selective auto-regressive model), were tested on the same dataset, obtained AUC of 0.86 and 0.84, respectively (Those values were obtained from https://causeme.uv.es/rank/ for the same dataset). Those methods are VAR-based and fit linear models on the data, regressing present on past values and inspecting the regression coefficients to decide whether one variable is a Granger-cause of another. Our approach is optimized to deal with oil field related data, and we obtained good performance in this kind of datasets. Furthermore, we did not optimize our algorithm to the climate dataset in order to have a more general solution.

### Synthetic oil production field

We tested our algorithms in a synthetic oil field production dataset known as UNISIM-II-M-CO (see “Data availability” section). This dataset represents a typical Pre-Salt field in Brazil^43^ (for more information on the Pre-Salt, refer to “Actual oil production field” section).

**Figure 10:**
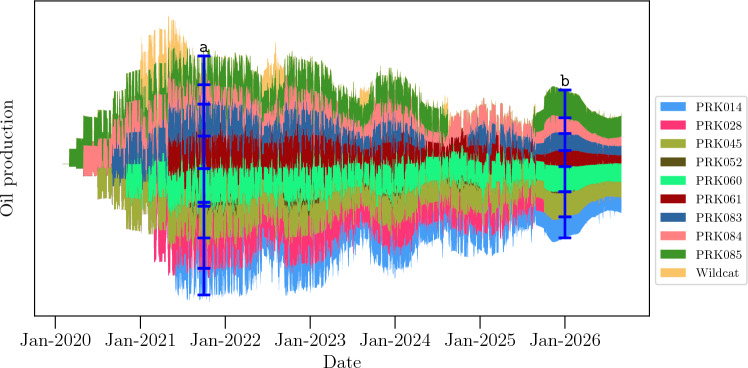
Oil production rate representation for the UNISIM-II model. Wells PRK052 and the Wildcat had only short operational periods. This kind of data visualization (called *streamgraph*) is meant to show the existence/absence of data over time, each time series represented in the plot has been normalized. *To interpret the plot*: Given a specific date on the x-axis, the vertical thickness of each time series indicates the relative oil production for each producer compared to the others. For instance, at timestamp labeled as a, there were nine producers active and that the producer PRK052 had the lowest production rate. Similarly, at timestamp labeled as b, there were seven active producers, PRK028 was closed and PRK014 had the highest production rate at that time. This methodology applies to each figure utilizing *streamgraphs* to visualize data.

**Figure 11:**
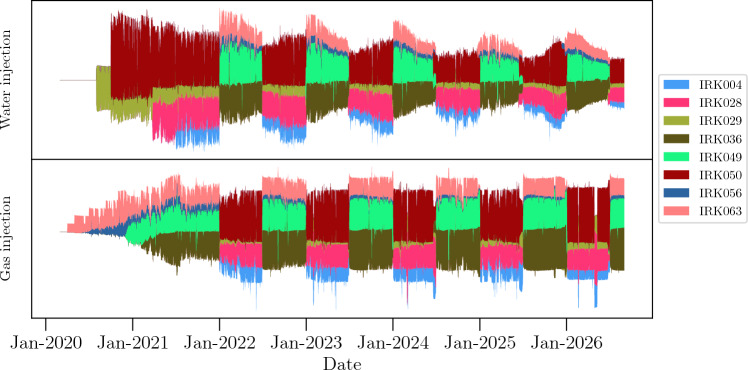
Injection data representation for the UNISIM-II field/model case. The injection is cyclical, i.e., every 6 months each injector switches the injected fluid from water to gas or vice-versa.

**Figure 12:**
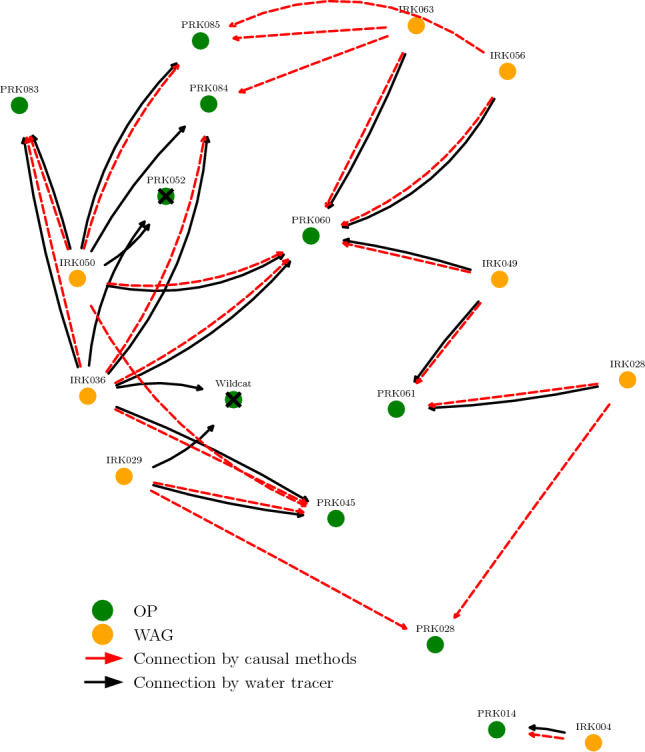
Connectivity map based on causality analysis applied to the UNISIM-II dataset. Connections detected by causality analysis are shown (red arrows) and compared against connections confirmed by water tracer (black arrows). The cross mark represents wells not included in the analysis due to their long shut-in period during the selected time window. In this dataset, our proposed method found connections result in a sensitivity of 0.74 and a specificity of 0.90.

This dataset simulates a reservoir with ten producer wells: PRK014, PRK028, PRK045, PRK060, PRK061, PRK083, PRK084, PRK085, and Wildcat; and eight injector wells: IRK004, IRK028, IRK029, IRK036, IRK049, IRK050, IRK0I15, and IRK063. The first letter of the well name indicates if it is a producer (P) or an injector (I). The oil production history is shown in Fig. 10, while the fluid injection history is depicted in Fig. 11. These plots are based on the *streamgraph* visualization technique^44^ to represent the existence or absence of data over time for each variable. The *y*-axis values cannot be interpreted as real values of material volume (as the data were transformed before plotting). As the wells PRK052 and Wildcat in Fig. 10 operated only for short periods, we excluded them from the analysis, resulting in Fig. 12. Additionally, since the injection data in Fig. 11 presents Water Alternating Gas (WAG) cycle of 6 months, we chose a 6-month time window for the causality analysis.

For this oil field related time series, we use producers’ BHP as targets. The input for the analysis include the producers’ past BHP and the combination of BHP and fluid injection rate for each injector, taking into account whether water or gas was being injected based on the corresponding cycle. This information follows the pipeline depicted in Fig. 4. The results are summarized in Fig. 12 and compared with connections confirmed by tracer information.

In an oil field, tracers (chemical compounds) can be injected into the reservoir to track the movement of the fluids^45^ between wells underground. After some time, the chemical tracers which are injected into the injection wells reach some producers and, from this information, injector–producer connections are confirmed. Those connections based on tracer detection are considered our ground truth. However, it’s important to note that the absence of tracer confirmation does not necessarily mean that the connection does not exist. It could simply mean that the tracer has not reached the producer yet. This applies to both simulated and real datasets. As seen in Fig. 12, there is a good agreement between the connections confirmed by tracer and the ones detected by our causal methods. We highlight that our methodology is able to detect connections not yet confirmed by tracer, such as the ones to PRK028 from injectors IRK029 and IRK028. These connections are identified based on pressure communication and may exist due to the injectors-producer proximity.

The lag dependence between cause and effect, i.e., the time between injection and its impact on the producers, is in the order of days, typically ranging from 1 to 6 days. However, when examining the travel time obtained from the tracer data for the simulated dataset, we observed no clear trend between travel time (difference between the time of detection at the producer and time of injection in the injector) and interwell distance. The complexity of the subsurface geology within the reservoir can lead to preferential pathways that make interwell connections faster regardless of the distance (for example fault pathways, fracture corridors or other high-permeability features/layers in the reservoir formation). As a result, the lack of a clear trend in the travel time poses challenges in validating the lags we estimated from the causal analysis.

### Actual oil production field

Addressing these challenges and effectively managing the Pre-Salt field requires advanced technological solutions, innovative approaches, and comprehensive understanding of the complex reservoir characteristics. The use of data-driven methods, such as the ones we employed in our analysis, can contribute to enhancing production optimization, reservoir management, and overall field performance, even in the case of heterogeneous reservoirs like the Pre-Salt field. 

We applied our methods to a real/private dataset obtained from a Pre-Salt field in Brazil. The Brazilian Pre-Salt is a significant offshore petroleum reserve localized in ultradeep water ($\sim$ 3000 m-depth) off the coast of Brazil. It is regarded as one of the most promising oil discoveries in recent years. It is characterized by thick and high-quality oil-bearing rock formations beneath a thick layer of salt. The big challenge for Brazilian Pre-Salt field development is due to the complex geology, the distance from the coast, the presence of variable $\hbox {CO}_{2}$ content in the reservoir, water depth, reservoir depth, salt layer, and flow assurance issues.

In the Brazilian Pre-Salt field, oil is extracted from reservoir rocks located deep beneath seabed. Specialized drilling rigs are used to drill wells that penetrate through the salt layers, reaching the oil reservoirs. The oil is then extracted using subsea pumps or natural pressure. Subsea production systems and Floating Production Unit (FPU) enable the extraction and processing of the oil, which is then transported to refineries.

**Figure 13:**
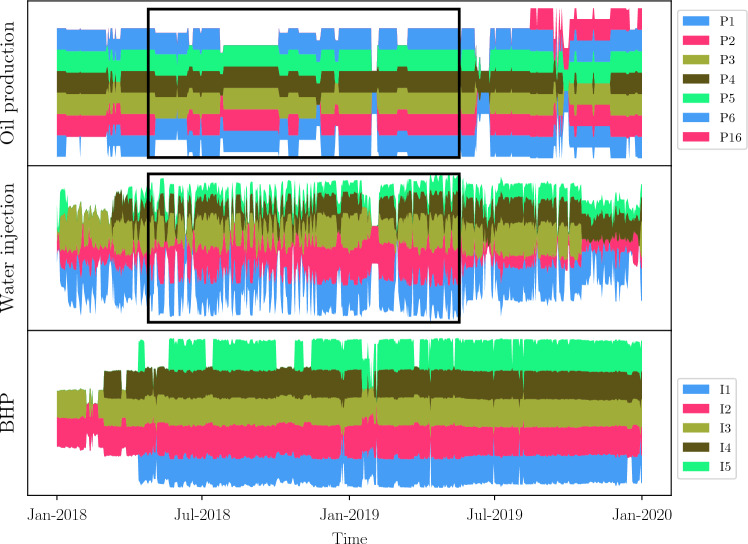
Visualization of available data for the low region of the Pre-Salt oil production field. The first panel represents data coming from the producers and the remaining panels represent data coming from injectors.

**Figure 14:**
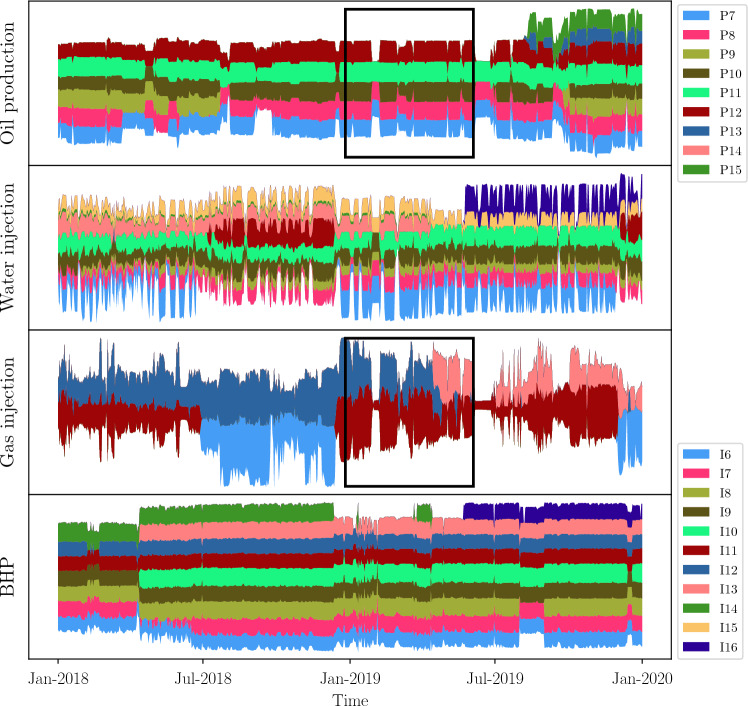
Visualization of available data for the high region of the Pre-Salt oil production field. The first panel represents data from producers, the remaining panels represent data coming from injector including injection BHP.

This Pre-Salt field consists of 16 production wells and 16 injection wells. The entire field is split into two regions, low and high, based on the wells’ location. Figures 13 and 14 show a visual representation (not the real values) of both oil production rates (upper panel of each plot) and injection history (water injection rates, gas injection rates, and injection BHP) over time. This representation allows us to see when a particular well was open (producing or injecting) or closed. The low region of Fig. 13 has fewer producers and injectors than the high region, making it easier to find a time window in which every well operates. In the high region of Fig. 14, there are more wells and the dynamics of injection (three bottom variables) frequently change, making it harder to pick up a time window.

To choose the time windows to apply our algorithms, the main criterion is to guarantee that most of the wells (both producers and injectors) per region were active (i.e., open), had available data and, in the case of well closure, that it was for a short period. In the real field data, the daily oil rates are apportioned (measurements are not made individually by well), so we chose to use daily BHP data for producers (since those measurements are made per well) and either water or gas injection rate together with injection BHP for the injectors. Therefore, we did choose the wells P1, P2, P3, P4, P5, P6, I1, I2, I3, I4, and I5 for the low region and the selected time series spans over a period of $\sim$ 1 year. In Fig. 13, we show the chosen time window for analyzing the wells in the low region. For the high region, the chosen wells were P7, P8, P10, P11, P12, I6, I7, I8, I9, I10, and I11. The chosen time window spans over a period of $\sim$ 6 months (see Fig. 14). Because there are more wells than those in the low region, periods of opening/closing (see Fig. 14, the upper panel that shows oil production history) are more diverse, as well as the injection cycles for the injector wells. Consequently, it is harder to choose a larger time window with more wells operating and, in the case of injectors, without switching the injection cycle within the time window. This led us to use a $\sim$ 6-month time window for the high region. No communication between the high and the low regions is expected, according to the reservoir properties, data and spatial distribution of the wells.

**Figure 15:**
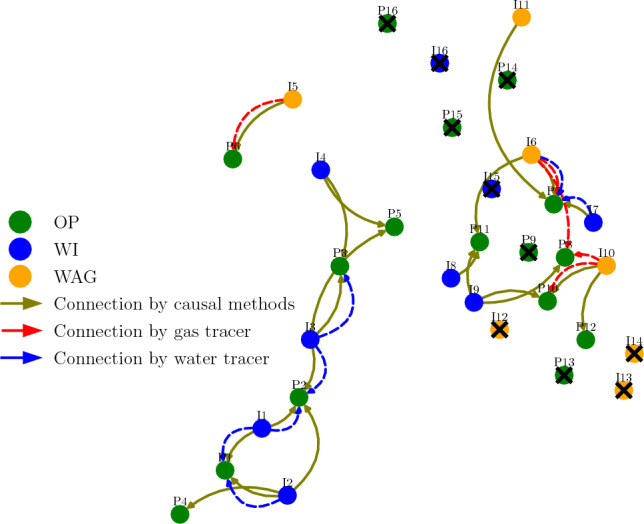
Connectivity map based on causality analysis applied to the Pre-Salt oil production field. The location of each well is shown and so are established connections based on our method and on tracer data. The cross mark represents wells not included in the analysis.

A connectivity map of the field is shown in Fig. 15. In this real field, there are Oil Production (OP), WAG and Water Injection (WI) wells. The interwell connections confirmed by either water or gas tracers are plotted as well as the connections detected by our causality method. Anyhow, it is worth noticing that not every OP monitors for tracers. Comparing the connections, we see a good agreement between detected and confirmed connections, only one confirmed connection is missed: to well P8. The behavior within an oil reservoir is highly dynamic, changing in time scales from months to years depending on whether new producers or injectors are drilled. It could happen that, within the chosen time frame, connections to P8 are not strong enough to be detected.

It could be misleading to try to quantify the agreement between detected connections by causality and confirmed connections by tracer data through metrics like TPR and FPR, or similar metrics. As stated, we do not know every existing connection in the reservoir to use as our ground truth and perform comparison.

The estimated lags are within a 1–8 day range, as we use pressure-related time series from both injectors and producers, and we expect the communication to be faster by pressure. It is not possible to validate those results due to the lack of sufficient datapoints from tracer information for this real dataset.

## Conclusions and future work

This paper showed our approach to establishing causal relationships among time series. The analyses were performed on three datasets: one climate and two oil field related data sets. We performed successful *causal discovery* aiming to estimate which connections exist without quantifying the contribution of each driver in the target it causally affects.

Our approach is data-driven, and we leverage the advantages of ML models that provide the importance of each feature used as input. We use this feature’s importance to determine the target’s current value-lagged dependence. We also devised a general pipeline to establish causal relationships that can be applied to time series related to different fields.

The proposed solution goes beyond correlations in an attempt to unveil the system’s underlying dynamics and the relationships’ directionality. Despite the difficulties of having short time windows to work with, we extracted valuable insights regarding interwell connectivity and validated them with the tracer data in the oil field related datasets. Not every interwell connection is confirmed by tracers; therefore, in this scenario, we also depend on the opinion of reservoir engineering specialists to validate our findings. Ideally, it is desirable to have tracer confirmation for every plausible (closed well pairs) connection, however using tracers has an associated economic and operational investment in the applied tracer chemical, equipment, and specialists (involving reservoir engineers, chemists, and field specialists). To our knowledge, this is the first study in the area of oil field production that establishes interwell connectivity based on causal analysis of production data. Understanding and proving the interwell connectivity has a vital impact on reservoir management.

One of the main difficulties we faced was related to the similarity among some time series from different sources (specifically, different injectors). To overcome this, we proposed to combine the time series coming from the same source into one and use this output as a predictor. This solution worked well for the oil field related datasets, providing a way to differentiate time series from different sources.

Future work comprises performing *causal inference*, which allows us to compute the strength of each connection. This is particularly important in the oil field application area because quantifying the strength of the injector-producer connection allows for more fine-grained planning of new strategies of injection or production, depending on the quantification of established connections and gives an independent way to validate connections and use these to improve 3D reservoir models. In this way, we provide an alternative to quantifying the strength of the connection; this quantification is traditionally made using Capacitance-Resistance Model (CRM). In the same field of study, we want to consider the producer-producer relation in our approach.

Another future work is to improve the performance of our approach when considering different applications.

Additionally, another research front is to perform *counterfactuals* analysis, i.e., test what would happen if changes are made to the driver’s data: Does the connection to the target hold? Does the strength change? The overall goal is to provide interpretability to some of our ML-based causal algorithms.

## Data Availability

Both the datasets for climate and UNISIM-II, used in this paper, are available for public access: *Climate dataset*: Data downloaded from the platform CauseMe.net. The dataset is available at https://causeme.uv.es/model/TestWEATHnoise/ (registration required). *UNISIM-II dataset*: Available by UNISIM group at the University of Campinas at https://www.unisim.cepetro.unicamp.br/benchmarks/en/unisim-ii/overview.
